# Synergistic interactions between glycogen and trehalose mediate adaptation to the stationary phase in *E. coli*

**DOI:** 10.1128/jb.00544-25

**Published:** 2026-04-22

**Authors:** Nicolaus A. Jakowec, Manat Chopra, Melissa Finegan, Steven E. Finkel

**Affiliations:** 1 Molecular and Computational Biology Section, Department of Biological Sciences, University of Southern California, Los Angeles, California, United States; National Institutes of Health 2511, Bethesda, Maryland, USA

**Keywords:** metabolic regulation, glycogen, trehalose, long-term survival, stationary phase

## Abstract

Glycogen and trehalose are multifunctional carbohydrates that contribute to bacterial adaptability, stress tolerance, and energy homeostasis, yet the mechanisms underlying these characteristics are not fully elucidated. In *Escherichia coli*, we reveal a metabolic and regulatory coupling between glycogen and trehalose with the global flux-sensing regulators cAMP-CRP and Cra during the stationary phase. Using mutants lacking glycogen synthase (*glgA*), trehalose-6-phosphate synthase (*otsA*), or both, we measured carbon storage, competitive fitness, stress sensitivity, expression of flux-sensing target genes, and concentrations of ATP, reactive oxygen species, and advanced glycation endproducts. Single mutants showed reduced fitness, while the *glgA otsA* double mutant exhibited transient competitive advantages in early stationary phase but severe stress sensitivity and ultimate fitness collapse. The double mutant had the lowest ATP, yet elevated redox activity, indicative of uncoupled metabolism. Transcriptional analysis revealed upregulated *pfkA* and *ptsG* and downregulated *pykF* and *rpoS*, consistent with Cra inactivation and cAMP-CRP activation. Wild-type cells increased glycogen and trehalose storage in response to glucose stress, while the *glgA otsA* mutant showed severe glucose stress toxicity, demonstrating that both compounds act as glucose buffers; the antioxidant carnosine partially rescued the fitness of all strains. These results suggest that glycogen prevents fructose-1,6-bisphosphate accumulation that inappropriately inactivates Cra, while trehalose recycling maintains PTS flux, preventing aberrant cAMP-CRP activation. Loss of both causes dysregulated flux-sensing where transcriptional programs misalign with metabolic state, leading to overflow metabolism, methylglyoxal accumulation, and fitness collapse.

**IMPORTANCE:**

Bacteria in nature endure prolonged energy limitation interspersed with nutrient influxes— termed the “feast-famine” lifestyle. To cope with environmental scarcities and fluctuations, bacteria manage limited energy reserves and coordinate metabolic programming with external conditions and intracellular demands. This study reveals how *Escherichia coli* manages carbon storage during starvation through glycogen and trehalose, working in a functionally specialized yet synergistic partnership. Critically, these pathways are interconnected with global transcriptional regulatory systems—cAMP-CRP and Cra—coordinating nutrient scavenging, stress responses, and energy metabolism. Disrupting both pathways dysregulates these regulators, causing bacteria to initially outcompete wild-type cells through enhanced resource acquisition, but ultimately compromising long-term survival as stress resistance is impaired. Understanding stationary phase physiology is essential because this growth-arrested state characterizes many natural habitats.

## INTRODUCTION

Glycogen and trehalose are glucose-derived polysaccharides and disaccharides, respectively, that are synthesized endogenously across a wide variety of organisms, including insects, fungi, and bacteria (1–3). In bacteria, both compounds contribute to cellular survival and pathogenicity but differ in their physiological roles (4, 5). Glycogen primarily serves as a carbon reserve and a carbon shunt to maintain metabolic homeostasis (6), while trehalose acts as a stress protectant with additional emerging metabolic and regulatory roles (7–10). Understanding the metabolic relationship between these two pathways can provide insights into compensatory strategies and functional redundancies that may serve as necessary mechanisms for survival under conditions of stress, including limited nutrient resources. Furthermore, how glycogen and trehalose influence survival and fitness in the fluctuating, energy-limited environments that bacteria frequently encounter in nature is poorly understood. The carbon-limited batch culture condition known as long-term stationary phase (LTSP) recapitulates this “feast-famine” lifestyle and offers an experimental framework to explore how glycogen and trehalose mediate adaptive homeostasis (11, 12).

**Fig 1:**
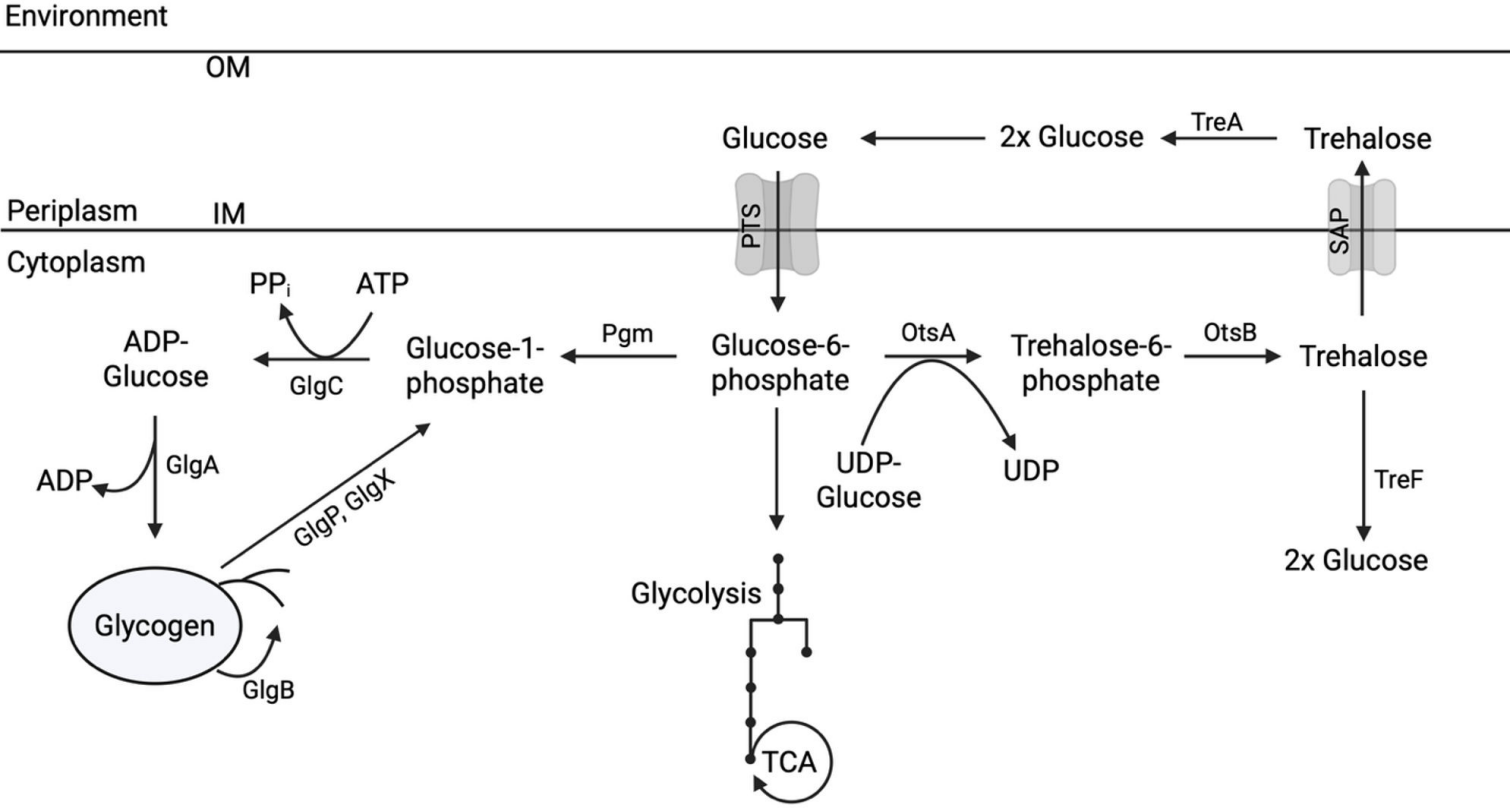
Overview of glycogen and trehalose metabolism in *E. coli.* Glucose enters the cytoplasm and is concomitantly phosphorylated by the phosphotransferase system (PTS), converting it to glucose-6-phosphate. In addition to entering central carbon metabolism (CCM), glucose-6-phosphate also serves as the primary precursor for glycogen or trehalose synthesis. For glycogen synthesis, phosphoglucomutase (PGM) catalyzes the conversion of glucose-6-phosphate to glucose-1-phosphate, which is then condensed with ATP by the enzyme GlgC to form ADP-glucose. GlgA catalyzes the transfer of glucose units from ADP-glucose to a growing glycogen chain as alpha-1,4-linkages, which can be converted to branching alpha-1,6-linkages by GlgB. The debranching enzyme GlgX and the glycogen phosphorylase GlgP facilitate glycogen breakdown into glucose-1-phosphate. OtsA catalyzes the first step in trehalose production, converting glucose-6-phosphate and UDP-glucose into trehalose-6-phosphate. OtsB removes the phosphate from trehalose-6-phosphate, forming free trehalose. In the cytoplasm, trehalose can be hydrolyzed into glucose by TreF. Alternatively, trehalose excreted into the periplasm via stretch-active porins (SAPs) can be broken down into glucose by TreA, and the liberated glucose can be imported back into the cytoplasm via the PTS (OM = inner membrane, IM = inner membrane).

Glycogen is a highly branched polysaccharide consisting of glucosyl residues that are linked by α-1,4-glycosidic bonds in linear chains and α-1,6-glycosidic bonds at branching points (13). In *E. coli*, glycogen metabolism is traditionally thought to be triggered when cells transition from logarithmic growth to stationary phase or when bacteria experience limited nutrient availability, such as during nitrogen limitation with sufficient carbon (14, 15). The glycogen biosynthetic enzymes are encoded by the genes *glgC* (ADP-glucose pyrophosphorylase), *glgA* (glycogen synthase), and *glgB* (glycogen branching enzyme); the catabolic enzymes are encoded by *glgX* (glycogen debranching enzyme) and *glgP* (glycogen phosphorylase) (16) (Fig. 1). These five genes are organized into a single operon, *glgBXCAP*, with an alternative promoter within the *glgC* gene that can direct *glgAP* expression (17). This dual promoter system enables co-expression of all five enzymes and reconstitution of the entire glycogen metabolic pathway (15, 18, 19).

After glucose-6-phosphate is converted to glucose-1-phosphate by phosphoglucomutase, the enzyme GlgC catalyzes the synthesis of ADP-glucose from glucose-1-phosphate and ATP. GlgA catalyzes the transfer of the glucosyl unit of ADP-glucose to a glycogen primer to form an α-1,4-glycosidic bond (18, 20). Following glycogen chain elongation, GlgB creates branches in the glycogen molecule by introducing α-1,6-glycosidic linkages (21), which can be hydrolyzed by GlgX. GlgP facilitates glycogen breakdown into glucose-1-phosphate by removing glucose units from the nonreducing ends (19).

Trehalose is a nonreducing disaccharide comprised of two glucose units linked via an α,α-1,1-glycosidic bond. Trehalose can be acquired from the environment or synthesized endogenously (22). Synthesis in microorganisms is induced in response to various external stresses, particularly cold shock, osmotic stress, and heat shock, as well as during the transition to the stationary phase (22–24). Due to the disaccharide’s unique physicochemical properties—nonreducing nature, high glass transition temperature, and ability to stabilize biomolecules—trehalose can protect proteins and cell membranes from damage from a variety of stresses, including oxidative stress and osmotic stress (25, 26). In *E. coli*, the trehalose biosynthetic enzymes OtsA (trehalose-6-phosphate synthase) and OtsB (trehalose-6-phosphate phosphatase) catalyze a two-step conversion of glucose-6-phosphate and UDP-glucose into trehalose (Fig. 1). Trehalose breakdown back into glucose is facilitated by two trehalase enzymes: TreA in the periplasm and TreF in the cytoplasm. Trehalose can be secreted into the periplasm, where it is hydrolyzed by TreA into two glucose molecules, which can be transported back into the cytoplasm as glucose-6-phosphate via the glucose-specific PTS, with phosphoenolpyruvate (PEP) as the phosphoryl donor (27, 28).

Although trehalose and glycogen metabolism have been studied independently in *E. coli*, their potential interrelationship has not been directly examined. Both biochemical pathways draw from glucose-6-phosphate (G-6-P) as a primary precursor, and their biosynthetic and catabolic genes are transcriptionally regulated by the stationary phase-specific sigma factor RpoS (24, 29). Previous work demonstrated that loss of TreA alters cAMP–CRP signaling via modulation of the PTS phosphorelay, suggesting that trehalose metabolism can influence global transcriptional regulation (8). The shared use of G-6-P as a precursor and similar gene regulation prompted us to investigate whether trehalose and glycogen serve not only parallel but also co-regulating roles in stress adaptation and metabolic homeostasis. To dissect the roles of glycogen and trehalose during dynamic physiological adaptations of *E. coli*, we subjected mutant strains impaired in glycogen and/or trehalose biosynthesis to the carbon-limited batch culture condition known as long-term stationary phase (LTSP) (11).

We hypothesized that glycogen and trehalose flux together play a role in the coordination of survival strategies during the stationary phase, with trehalose potentially influencing energy balance and glycogen affecting stress resistance systems. To test this, we generated mutant strains lacking biosynthetic enzymes for glycogen (*glgA*), trehalose (*otsA*), or both (*glgA otsA*), and assessed their physiological responses under LTSP conditions. LTSP occurs under batch culture conditions of LB medium and mimics natural feast-famine cycles where cells experience nutrient depletion, oxidative and glycation stress, and intra-population competition (11, 30–32). This culture system allows for the investigation of carbon storage, stress survival, and regulatory rewiring under dynamic, energy-limited conditions. Through competition assays, metabolite quantification, and gene expression analysis, we evaluated the impact of glycogen and trehalose deficiency on fitness and stress resilience.

## MATERIALS AND METHODS

### Bacterial strains and mutant construction

All strains used in this study are isogenic and derived from the W3110-lineage *Escherichia coli* K-12 strain ZK126 (Δ*lacU169 tna-2*) (33). Isogenic *otsA*, *glgA*, and *glgA otsA* null mutant strains were constructed via bacteriophage P1 transduction into ZK126 using a donor strain carrying the respective mutant gene replaced with a kanamycin resistance (Kan^R^) allele obtained from the Keio collection (34). Replacement of the gene of interest with the kanamycin-resistance cassette was confirmed by PCR (data not shown). For the generation of the double knockout mutant strain *glgA otsA*, the Kan^R^ cassette in mutant strains was excised using the pCP20 FLP recombinase plasmid. The FLP plasmid was cured by growth at 43°C, and cassette removal was confirmed by PCR. Additional gene knockout strains were constructed by another round of bacteriophage P1 transduction for the introduction of the second Kan^R^ cassette (35). ZK1142 is a nalidixic acid-resistant isogenic wild-type (WT) strain derived from ZK126 (36, 37). Previous work has shown that the point mutation leading to nalidixic acid resistance is neutral in long-term stationary phase batch culture experiments (8, 11, 30, 32, 33, 36–38).

### Culture conditions, media, and titering assays

For both monoculture and coculture experiments, strains were incubated in 5 mL LB (Lennox) broth (10 g/L tryptone, 5 g/L yeast extract, and 5 g/L NaCl; Difco, Franklin Lakes, NJ) in 18 × 150 mm borosilicate test tubes at 37°C with aeration in a TC-7 roller drum (New Brunswick Scientific, Edison, NJ). Viable cell counts were measured using the spot titering assay with a limit of detection of ≥1,000 CFU/mL. Cells were plated on LB agar for monoculture experiments or on LB agar with nalidixic acid (20 μg/mL) or kanamycin (50 μg/mL), as appropriate, for coculture experiments.

### Stress assays

For heat stress assays, overnight stationary phase cultures were shifted to a water bath at a temperature of 53°C. Viable-cell counts were periodically determined. For oxidative stress assays, 450 mM of H_2_O_2_ was added to stationary phase cultures, and viable counts were periodically determined over an hour. For glucose stress cultures, glucose was added to cultures to a final concentration of 0.4% (wt/vol) at inoculation, and viable counts were determined daily. All assays were performed in triplicate, and standard deviation was determined.

### Advanced glycation endproduct ELISA assay

Cells from entire 5 mL cultures were pelleted, washed with PBS, and resuspended in 500 µL 10mM Tris-HCL (pH 7.5) and PBS. Cells were sonicated on ice using a Branson Sonifier 250 (Emerson) set to maximum setting, six times for 10 s with 30-s rest, followed by enzymatic digestion with DNase I (50 μg/mL), RNase A (50 μg/mL), and lysozyme (20 μg/mL) at 37°C for 1 h. Protein concentration of the clarified lysate was quantified using the Quick Start Bradford dye reagent, following the manufacturer’s instructions (Bio-Rad). 100 μL of protein extract, at concentrations ranging from 0.5 to 1.0 mg/mL, was added to an AGE conjugate-coated plate as part of an OxiSelect AGE Competitive ELISA Kit (Cell Biolabs). Endogenous AGEs were quantified according to the manufacturer’s instructions using an AGE standard curve, and AGE levels were normalized to protein concentration.

### Batch culture competition assays

Direct competitions between the parental and mutant strains were performed using cells from overnight cultures of both strains co-inoculated at 1:1,000 (vol:vol) dilution into 5 mL LB cultures. The relative fitness of each strain was determined by plating cells on agar plates containing the appropriate antibiotics. Titering assays were performed daily over 10 days to quantify the viable counts of each strain throughout the stationary phase and LTSP. For carnosine supplementation experiments, 25 mM carnosine was added to cultures prior to inoculation.

### Glycogen and trehalose quantification

Quantitative analysis of glycogen and trehalose concentrations was modified from a protocol in Chen and Futcher (39). For each timepoint, 5 mL of cultures was pelleted, washed with 10 mM Tris-HCl (pH 7.5) in PBS, and resuspended in 125 µL of 0.25 M Na₂CO₃. Suspensions were then heated at 95°C for 3 h to lyse the cells. After heating, the pH of each sample was adjusted to pH 5.5 and the volume to 500 µL by the addition of 75 µL of 1 M acetic acid and 300 µL of 200 mM sodium acetate buffer (pH 5.2). Samples were vortexed vigorously to resuspend and disperse cell debris, and 100 µL aliquots were immediately distributed into four fresh 1.5 mL Eppendorf tubes.

For trehalose measurement, 3 µL of porcine trehalase (~2.3 U/mL; Sigma) was added to one aliquot of each sample, and tubes were incubated on a nutator at 37°C overnight. For glycogen measurement, a 20 mg/mL solution of *Aspergillus niger* α-amyloglucosidase (~70 U/mg) was freshly prepared in 0.2 M sodium acetate buffer (pH 5.2). Then, 5 µL of this solution was added to a separate aliquot from each sample, and tubes were incubated in a 57°C water bath overnight.

Following enzymatic digestion, supernatants were clarified by centrifugation, and glycogen and trehalose levels were quantified using a Glucose Assay (Hexokinase) Kit (Sigma) according to the manufacturer’s instructions. Liberated glucose from each reaction was compared to an untreated aliquot to correct for background glucose levels.

### ATP measurement, AlamarBlue assays, and reactive oxygen species quantification

ATP was measured using the BacTiter-Glo Microbial Cell Viability Assay Kit (Promega, Madison, WI) according to the manufacturer’s instructions. Briefly, 100 µL of a 24-h culture was mixed with 100 µL BacTiter-Glo reagent in an opaque 96-well plate. After 5 min at room temperature, luminescence was read on an Infinite 200 Pro Microplate Reader (Tecan, Baldwin Park, CA). Luminescence (relative luminescence units [RLU]) values were normalized to the WT strain, which was given a value of 1.0.

Metabolic activity was quantified using AlamarBlue (Thermo Scientific) according to the manufacturer’s absorbance-based protocol. Twenty microliters of AlamarBlue reagent was added to 180 µL of 24-h cultures in a clear 96-well plate. The plate was covered and incubated at 37°C for 1 h. Absorbance was then measured at 570 nm and 600 nm using an Infinite 200 Pro Microplate Reader (Tecan, Baldwin Park, CA). A filtered supernatant-only control was included to correct for background absorbance. Percent reduction of AlamarBlue, corresponding to metabolic activity, was calculated using the manufacturer’s formula:


$$\%\text{reduction}=\frac{{\left(\varepsilon_{OX}^{600}A_{570}-\varepsilon_{OX}^{570}A_{600}\right)}_{\text{sample}}}{{\left(\varepsilon_{RED}^{570}A_{600}-\varepsilon_{RED}^{600}A_{570}\right)}_{\text{control}}}\times 100$$

where $\varepsilon_{OX}^{570}$, $\varepsilon_{OX}^{600}$, $\varepsilon_{RED}^{570}$, and $\varepsilon_{RED}^{600}$ are the molar extinction coefficients of oxidized and reduced AlamarBlue at the respective wavelengths (80,586, 117,216, 155,677, and 14,652 M⁻¹ cm⁻¹, respectively). Results were normalized to cell density (OD_₆₀₀_) to account for differences in biomass.

For measurement of intracellular reactive oxygen species (ROS) levels, 0.5 mL of stationary phase cultures at 24 h were pelleted, washed, and resuspended in 1 mL of PBS. 100 mM stock of 2′,7′-dichlorofluorescin diacetate (DCFH-DA) (Sigma) was made in DMSO and added to each sample for a final concentration of 500 µM. Cells in the presence of DCFH-DA were incubated at 37°C in the dark for 1 h and again washed and resuspended with PBS. Fluorescence was measured at excitation and emission wavelengths of 485 and 525 nm, respectively.

### Quantitative real-time PCR

5 μL aliquots of overnight cultures of wild-type ZK1142 or mutant strains were inoculated into 5 mL of LB broth and incubated at 37°C for 16 h to reach the stationary phase. 0.5 mL of each culture (all strains had comparable stationary phase cell densities) was then mixed with 1 mL of RNA Protect (Qiagen, Valencia, CA), vortexed, incubated at room temperature for 5 min, and centrifuged at 7,500 rpm for 10 min to pellet the cells, which were resuspended in lysis buffer (30 mM Tris-HCl, 1 mM EDTA, pH 8.0, 15 mg/mL lysozyme, and 10 μL proteinase K). Total bacterial RNA was extracted using the RNeasy Mini kit (Qiagen, Valencia, CA) according to the manufacturer’s instructions. RNA samples were treated with DNase I (Qiagen, Valencia, CA) to eliminate genomic DNA. 0.4 μg of total purified RNA was reverse transcribed using a qPCRBIO cDNA synthesis kit (PCR Biosystems, London, UK). Quantitative Real-Time PCR (qRT-PCR) was run for 40 cycles on an Eppendorf Mastercycler ep Realplex with a mix of 1 μL (10 ng) cDNA, 1 μL each of forward and reverse primers (10 μM stock), and 10 μL of SyGreen Mix Lo-ROX (PCR Biosystems, London, UK). The relative fold change was calculated using the 2^−ΔΔC^T method (40). The *ihfB* gene was used as an internal reference for data normalization (41, 42).

### Statistical analyses

All viable count growth and survival curves, competition experiments, and stress assays were performed independently at least three times. Metabolite quantification, ATP, AlamarBlue, and ROS assays were conducted at least two times independently. RNA isolation and qRT-PCR experiments were conducted two times independently. For all experiments, except coculture competition experiments and glucose stress experiments, three liquid cultures (*n* = 3) were used per strain or condition. For coculture competition experiments and glucose stress experiments, five cultures (*n* =5) were used per strain or condition.

To evaluate whether carnosine statistical supplementation alters competitive dynamics between wild-type and mutant strains, fold change values (WT CFU/mL divided by mutant CFU/mL) were calculated at each timepoint for each biological replicate. Fold change values were log_10_-transformed to satisfy ANOVA assumptions of normality and heterogeneity of variance. Two-way analysis of variance (ANOVA) was performed using the statsmodels package (v0.14.6) in Python 3.12 with the model: Log_10_ (Fold Change) ~ Day × Carnosine, where Day represents the nine sampling timepoints (categorical factor), and Carnosine represents the two treatment conditions (LB or LB + 25 mM carnosine). Type II sum of squares was used to evaluate main effects and interaction terms. The Day × Carnosine interaction term tests whether the effect of carnosine on competitive fitness varies across the time course. For all other pairwise comparisons, including competition assays without carnosine supplementation (WT vs. mutant strain at individual timepoints), stress assays, monoculture growth, qRT-PCR, metabolic assays, and metabolite quantifications, unpaired two-sided t-tests were used. Data visualization and statistical analyses were performed using RStudio (Version 2025.09.1+401) with tidyverse packages and Python 3.12 (matplotlib, scipy, pandas, statsmodels). Statistical significance was defined as *P* < 0.05.

## RESULTS

### Glycogen and trehalose metabolism are important for stationary phase survival

**Fig 2:**
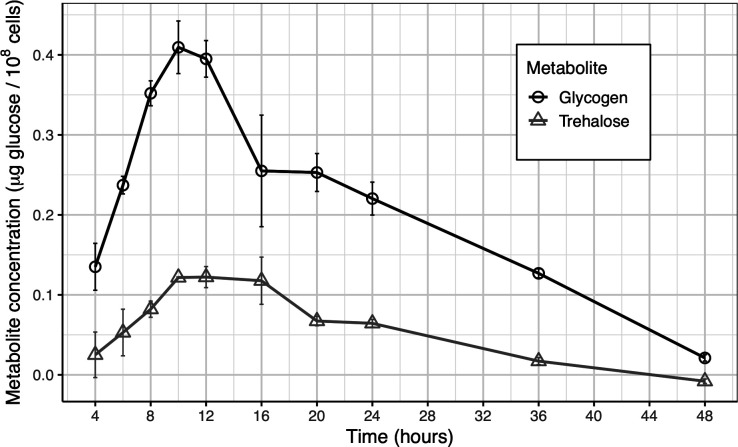
Temporal dynamics of glycogen and trehalose concentrations during the stationary phase. Intracellular glycogen (circles) and trehalose (triangles) levels in WT (ZK1142) *E. coli* in LB medium from 4 to 48 h post-inoculation. Data are mean ± s.d. (*n* = 3 cultures per timepoint).

In *E. coli*, glycogen and trehalose can accumulate as cells transition from exponential phase to stationary phase or encounter growth-limiting constraints (14, 24). The changes in concentration of either compound throughout the stationary phase are not well-studied. Furthermore, glycogen and trehalose levels have not been measured simultaneously in *E. coli* within the same batch culture experiment. To address this gap in knowledge, glycogen and trehalose levels were quantified in the wild-type strain (ZK1142) inoculated into LB medium over a 48-h period, at 10 different timepoints, from exponential to late stationary phase, to capture the full range of physiological conditions experienced by the microbes (Fig. 2). Glycogen and trehalose levels both increase from 4 h post-inoculation (exponential phase) until plateauing at ~10 h post-inoculation (stationary phase). While trehalose levels are largely unchanged from 10 to 16 h, glycogen levels decrease significantly from 12 h to 16 h. Trehalose levels decrease from 16 to 20 h, and both glycogen and trehalose levels gradually decrease from 24 h to 48 h (late stationary phase). At all timepoints measured, more glucose per cell was stored as glycogen than as trehalose.

**Fig 3:**
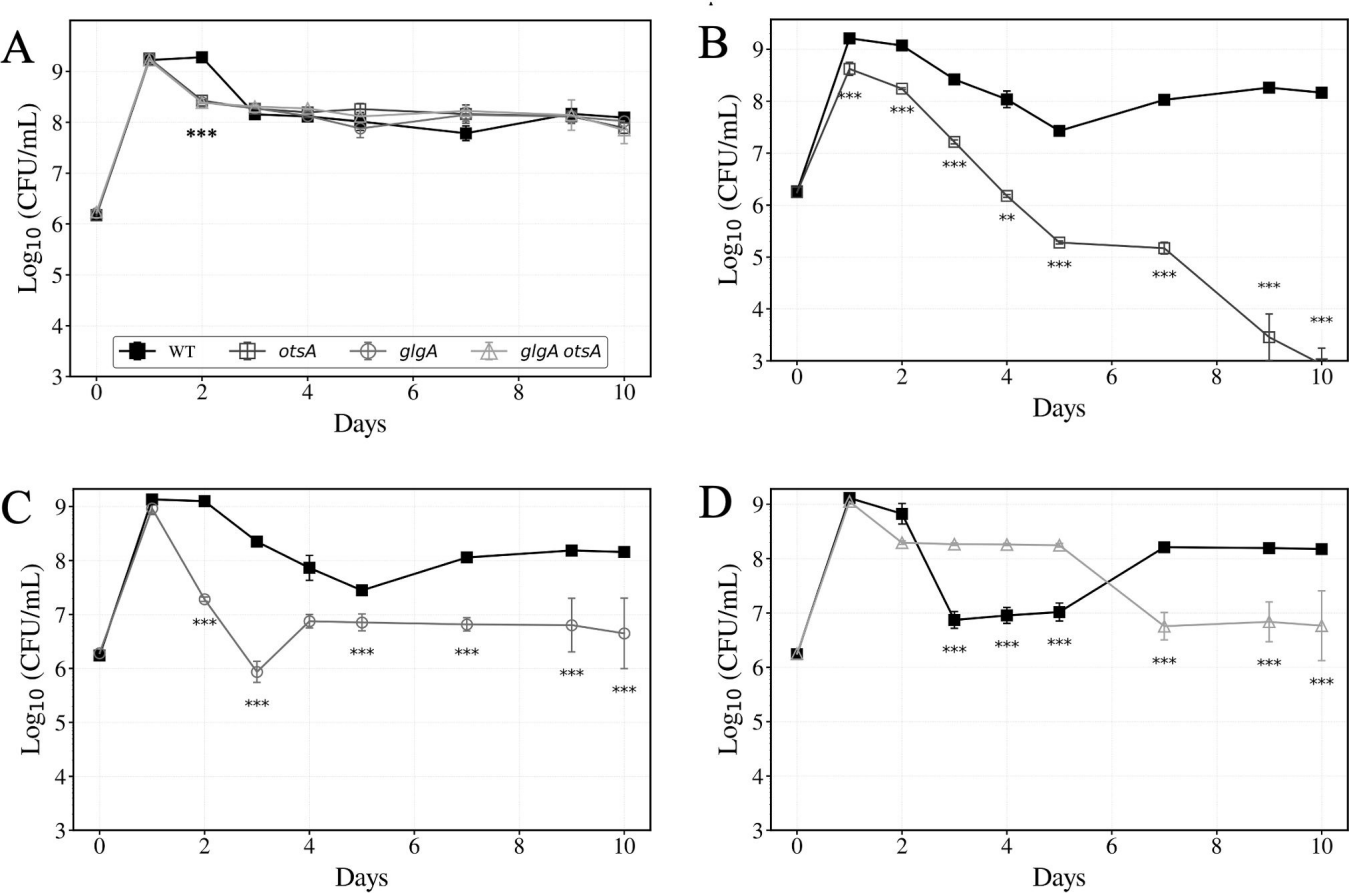
Viable cell counts of WT and mutant strains in LB medium. Black filled squares, WT; dark gray open squares, *otsA;* gray circles, *glgA*; light gray triangles, *glgA otsA*. (**A**) Monoculture growth curves of strains. *otsA*, *glgA*, and *glgA otsA* exhibit reduced stationary phase survival compared to WT. Asterisks below overlapping mutant lines at Day 2 indicate that each of the three mutant strains is significantly different from WT (***, *P* < 0.001 for each comparison; unpaired t-test; two-sided). (**B**) *otsA* vs. WT coculture competition. (**C**) *glgA* vs. WT coculture competition. (**D**) *glgA otsA* vs. WT coculture competition. Data are mean ± s.d. (*n* = 3 cultures per strain for monoculture, *n* = 5 cultures per strain for coculture competition; *, *P* < 0.05; **, *P <0.01*; ***, *P* < 0.001; unpaired *t*-test; two-sided).

Next, the impact of glycogen and/or trehalose synthesis on cell viability during the stationary phase and LTSP was assessed. *E. coli* strains with knockout mutations of *otsA*, encoding trehalose-6-phosphate synthase, *glgA*, encoding glycogen synthase, or a *glgA otsA* double mutant were inoculated into fresh LB medium, and viable cell counts were monitored daily. Following the stationary phase, *E. coli* cultures transition through the death phase until the viable cell density plateaus at approximately 10% of the stationary phase density, at which point survivors experience the batch culture condition of LTSP (11, 36, 43, 44). Strains were incubated in monoculture and viable cell counts were monitored (Fig. 3A). While WT and mutant strains reached comparable stationary phase cell densities at Day 1 (~1.8 × 10^9^ ± 0.4 × 10^9^ CFU/mL), the *otsA*, *glgA*, and *glgA otsA* mutant strains showed impaired survival relative to WT, all entering death phase and losing ~90% of viability 1 day earlier than WT. After entry into LTSP, all three mutant strains displayed similar viable cell counts to WT up to 10 days post-inoculation (9.6 × 10^7^ ± 4.9 × 10^7^ CFU/mL).

### Disrupting both glycogen and trehalose synthesis confers a transient competitive advantage during early LTSP

In addition to monoculture survival, competitive fitness was determined by coculturing each mutant strain with WT cells. Figure 3B shows that the *otsA* mutant yields on Day 1 are ~10-fold lower than the WT strain in coculture (WT, 1.6 × 10^9^ ± 2.8 × 10^8^; *otsA*, 4.2 × 10^8^ ± 3.4 × 10^8^, unpaired two-tailed *t*-test, *t* = 6.19, df = 8, *P* < 0.001). After Day 2, *otsA* mutant viability is reduced each day during LTSP relative to WT by ~10-fold, until day 5, when WT densities are higher by ~100-fold. From days 6 to 10, WT maintains this advantage, with *otsA* titers at **~**10⁵ CFU/mL, indicating limited recovery and continued competitive decline relative to WT.

Figure 3C shows that the *glgA* mutant reaches comparable day-1 yields relative to WT in coculture, yet has impaired fitness of ~100-fold on both day 2 and day 3 (day 3: WT, 2.24 × 10^8^ ± 8.56 × 10^7^; *glgA*, 8.64 × 10^5^ ± 6.02 × 10^5^, unpaired two-tailed *t*-test, *t* = 5.83, df = 8, *P* < 0.001). On day 5, WT viability is ~10-fold higher than *glgA*. From days 6 to 10, WT cell counts stabilize around 10⁷ CFU/mL, whereas *glgA* populations continue to decline.

While mutating either the *otsA* or the *glgA* genes impaired competitive fitness relative to WT, the *otsA glgA* double mutant displays a competitive advantage against WT cells in coculture (Fig. 3D). The WT and *glgA otsA* double mutant strain maintains similar cell counts through Day 2 of competition. However, from day 3 to day 5, the *glgA otsA* mutant titer counts are ~10-fold higher than WT. By days 6–10, this advantage diminishes as WT recovers and surpasses the double mutant, with *glgA otsA* titers declining more sharply after day 7. The biphasic competitive dynamics of the *glgA otsA* double mutant—exhibiting a competitive advantage over WT during early LTSP (days 3–5), followed by a fitness deficit compared to WT at later timepoints (days 8–10)—along with the competitive deficits observed in the *otsA* and *glgA* single mutant strains, were reproducible across independent experiments (Fig. S1).

### Disrupting trehalose synthesis increases sensitivity to heat and oxidative stress

**Fig 4:**
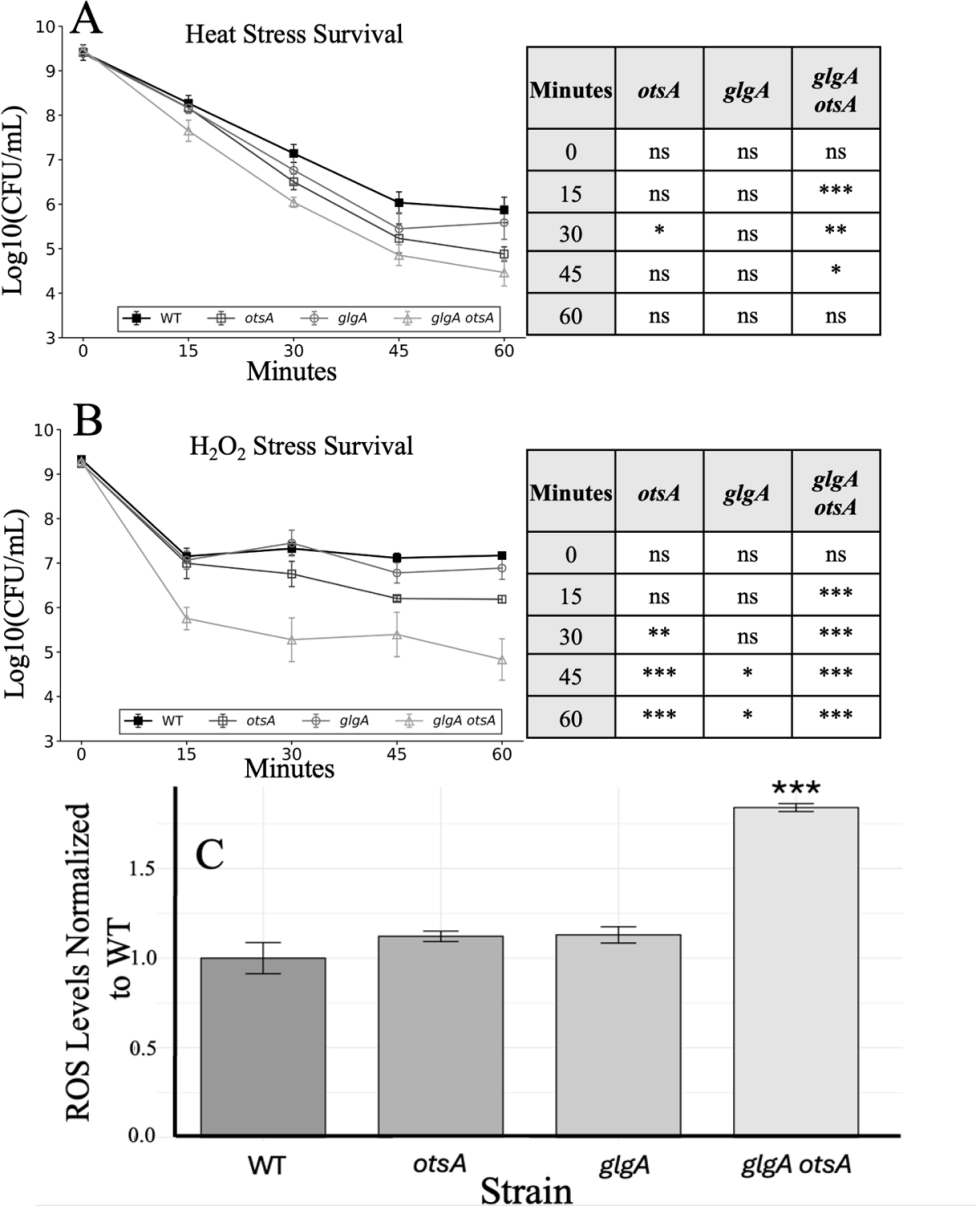
The *glgA otsA* mutant strain exhibits elevated stress sensitivity and ROS levels. (**A**) Stationary phase cultures were shifted to 53°C. Black filled squares, WT; dark gray open squares, *otsA;* gray circles, *glgA*; light gray triangles, *glgA otsA*. (**B**) Stationary phase cultures were treated with 450 mM H_2_O_2_. Black filled squares, WT; dark gray open squares, *otsA;* gray circles, *glgA*; light gray triangles, *glgA otsA*. For panels A and B, statistically significant timepoints are indicated in the accompanying tables: *, *P* < 0.05; **, *P* < 0.01; ***, *P* < 0.001; ns, not significant; unpaired t-test; two-sided. (**C**) ROS levels measured using 2′,7′-dichlorofluorescin diacetate (DCFH-DA) fluorescence. Data are mean ± s.d. (*n* = 3 cultures per strain; ***, *P* < 0.001; unpaired *t*-test; two-sided).

To elucidate the potential underlying mechanism of the competitive advantage of the *glgA otsA* double mutant versus WT, each of the mutant strains was subjected to a variety of stress assays, including heat stress, oxidative stress, and glycation stress. Stationary phase monocultures were subjected to heat stress at 53°C, and viable cell counts were determined at 15-min intervals for 1 h (Fig. 4A). While the *glgA* mutant strain exhibited heat sensitivity comparable to WT, the *otsA* and *glgA otsA* mutant strains showed increased viability loss. The *glgA otsA* strain was the most sensitive, displaying ~10-fold greater viability loss compared to WT.

To test for differential sensitivity to oxidative stress, hydrogen peroxide (H_2_O_2_) was added to stationary phase monocultures, and survival was measured (Fig. 4B). The *glgA* mutant strain showed slightly lower survival relative to WT 45 min after the addition of H_2_O_2_. The *otsA* mutant strain exhibited ~10-fold increased vulnerability to oxidative stress from 30 min onwards. The *glgA otsA* mutant displayed the highest sensitivity of any strain, showing ~100-fold less survival relative to WT from 15 min onwards. In addition to assessing H_2_O_2_ sensitivity, a cell-permeable ROS probe, 2′,7′-dichlorodihydrofluorescein diacetate (H2DCFDA), was used to quantify intrinsic ROS levels. It should be noted that DCFH-DA fluorescence is being used as a proxy for ROS and may not directly measure ROS levels but instead reports a broader array of oxidative stress conditions (45). While ROS levels of stationary phase cultures (LB medium without H_2_O_2_ supplementation) were similar between WT and the *otsA* and *glgA* mutant strains, ROS levels were significantly elevated in the *glgA otsA* mutant strain (Fig. 4C).

### Glycogen and trehalose synergistically protect against glucose stress

When cultures of LB medium are supplemented with glucose, glucose uptake can overwhelm the cell’s glycolytic pathway, resulting in metabolic bottlenecking and increased activity of side reactions (32, 45). These glucose stress reactions, referred to as overflow metabolism, can generate toxic byproducts, such as the reactive carbonyl metabolite methylglyoxal (MG) (46, 47). MG is a potent electrophile that is highly reactive with proteins, DNA, and lipids, where it nonenzymatically covalently modifies these compounds to form toxic molecules that accumulate over time and impair function, called advanced glycation endproducts (AGEs) (48, 49). AGEs and glycation stress also build up over time in LTSP, even in the absence of supplemented glucose, thus influencing long-term survival (32, 50). By acting as a carbon sink or as a stress protectant, glycogen and trehalose, respectively, have both been shown to mediate resistance to the glycation stress experienced under excess carbon conditions (51, 52). However, the potential synergistic relationship between glycogen and trehalose in response to glucose stress has not been explored, despite the compounds sharing glucose-6-phosphate as a common precursor.

**Fig 5:**
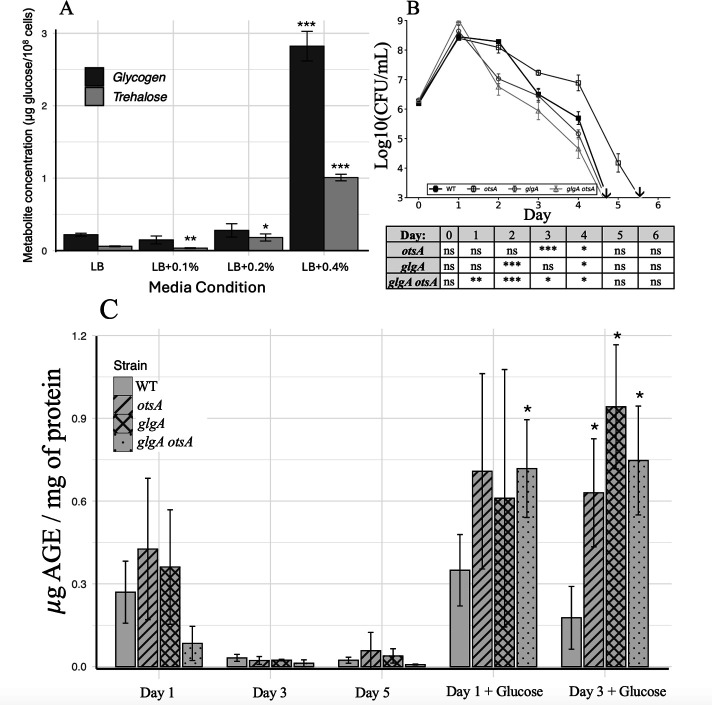
Glycogen and trehalose synergistically buffer against glucose stress. (**A**) Glycogen and trehalose quantification of WT stationary phase cultures (16 h post-inoculation) in LB medium and LB medium supplemented with increasing glucose concentrations (0.1%, 0.2%, and 0.4%). Data are mean ± s.d.(*n* = 3 cultures per strain or per condition. Statistical significance of glucose-supplemented conditions is compared to LB-only media condition: *, *P* < 0.05; **, *P <0.01*; ***, *P* < 0.001; unpaired *t*-test; two-sided). (**B**) Growth curve of strains in LB + 0.4% glucose. Black filled squares, WT; dark gray open squares, *otsA;* gray circles, *glgA*; light gray triangles, *glgA otsA*. (↓= below limit of detection, 10^3^ CFU/mL) Statistically significant timepoints are indicated in the accompanying table: *, *P* < 0.05; **, *P* < 0.01; ***, *P* < 0.001; ns, not significant; unpaired t-test; two-sided. (**C**) AGE ELISA. Data are mean ± s.d. (*n* = 3 cultures per strain or per condition; Statistical significance of AGE concentration of mutant strains is compared to AGE concentration of WT for the corresponding day/condition; *, *P* < 0.05; unpaired *t*-test; two-sided).

To assess how trehalose and glycogen levels respond to glucose supplementation, glycogen and trehalose concentrations were quantified in WT stationary phase cells after outgrowth under varying conditions of glucose supplementation, ranging from sub-toxic (0.1%) to toxic (0.4%) concentrations (Fig. 5A). While glycogen and trehalose accumulation 24 h post-inoculation declined slightly following the addition of 0.1% glucose, relative to an LB-only medium culture, trehalose accumulation increased when 0.2% glucose was added, while glycogen levels were not statistically different. Trehalose and glycogen levels both increased significantly (~10-fold each) following the addition of 0.4% glucose.

To assess differential sensitivity to glycation stress, mutant strains were incubated in LB medium supplemented with 0.4% glucose, and viable cell counts were measured daily (Fig. 5B). WT, *otsA*, and *glgA* strains similarly reached ~10-fold lower day-1 yields than when incubated in standard LB medium. Interestingly, the *glgA otsA* double mutant grew to a higher cell yield than any other strain in 0.4% glucose-supplemented LB medium. However, both the *glgA otsA* and *glgA* mutant strains’ viability decline sharply after Day 1, with Day 2 viability ~10-fold less than WT. While the *glgA* mutant strain has reduced viability relative to WT, the *glgA otsA* mutant strain shows more sensitivity to glucose stress than any other strain. Surprisingly, the *otsA* mutant strain exhibits increased survival relative to the WT strain during incubation under glucose stress, with viability ~10-fold higher than WT by Day 4. Independent biological replicates confirmed the reproducibility of these phenotypes, including the elevated day-1 cell yield of the *glgA otsA* mutant strain, enhanced *otsA* strain survival, and accelerated viability loss in the *glgA* and *glgA otsA* mutant strains (Fig. S2).

An ELISA was performed to quantify AGE accumulation in the strains under both standard LB and glucose-excess conditions (Fig. 5C). While AGE levels in all strains were comparable for all measured timepoints during LB incubation, the *glgA otsA* double mutant strain showed elevated AGE levels after 1 and 3 days of incubation in LB + 0.4% glucose, relative to WT. Both the *otsA* and *glgA* single mutant strains also accumulated greater amounts of AGEs after 3 days of incubation in LB +0.4% glucose.

### Supplementation with the antioxidant carnosine ameliorates fitness deficits of mutant strains against WT

To determine whether glycation stress and oxidative stress contribute to fitness differences between WT and mutant strains, competition cultures were supplemented with carnosine, a naturally occurring dipeptide (β-alanine + L-histidine) that possesses antioxidative and antiglycation properties (32, 53). Since carnosine reduces the oxidative stress and suppresses glycation, supplementation with carnosine could ameliorate the fitness deficits of mutant strains that are more susceptible to these stressors.

**Fig 6:**
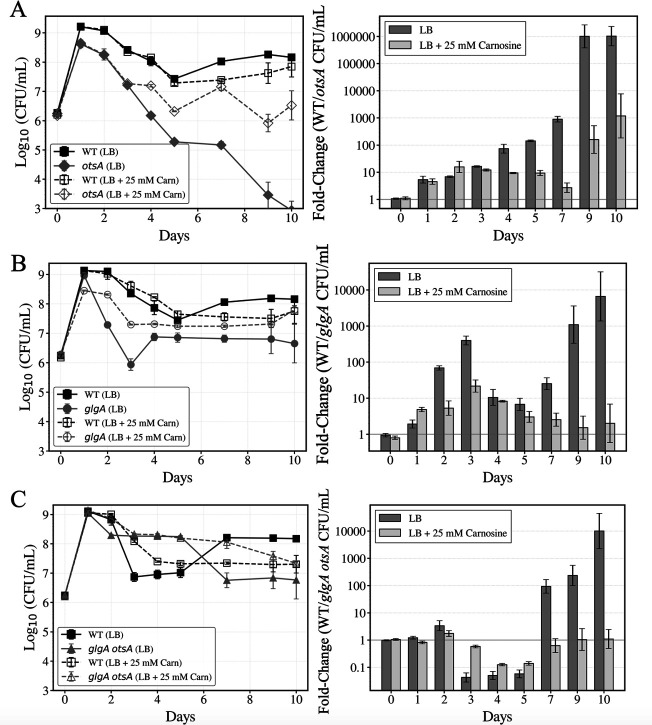
Carnosine supplementation provides time-dependent rescue of competitive fitness defects in glycogen and trehalose synthesis mutants. Competition experiments between wild-type and mutant strains in LB medium with or without 25 mM carnosine supplementation over 10 days. Left panels**:** Competition curves showing Log_10_ CFU/mL over time. Solid lines with filled markers represent LB medium; dashed lines with open markers represent LB + 25 mM carnosine. WT, black squares; mutant strains, gray markers: (**A**) *otsA* (diamonds), *glgA* (circles), and *glgA otsA* (triangles). Right panels: Fold-Change (WT CFU/mutant strain CFU) at each timepoint. Dark gray bars, LB; light gray bars, LB + 25 mM carnosine. A horizontal line at *y* = 1 indicates equal competitive fitness; values >1 indicate WT competitive advantage. Two-way ANOVA on log_10_-transformed fold change values testing the Day × Carnosine interaction revealed highly significant time-dependent effects of carnosine supplementation: (**A**) WT vs. *otsA* (F₈,₇₂ = 16.65, *P* = 1.10 × 10⁻¹³), (**B**) WT vs. *glgA* (F₈,₇₂ = 10.51, *P* = 1.22 × 10⁻⁹), (**C**) WT vs. *glgA otsA* (F₈,₇₂ = 15.63, *P* = 4.44 × 10⁻¹³), demonstrating that carnosine’s protective effect changes significantly over time. Data are mean ± s.d. (*n* = 5 biological replicates).

To quantitatively assess competitive dynamics, fold change values were calculated as the ratio of WT CFU/mL to mutant CFU/mL at each timepoint, where values greater than 1 indicate WT competitive advantage. Consistent with the hypothesis that mutant strains experience elevated oxidative and glycation stress, supplementation with 25 mM carnosine reduced the fold change (competitive advantage) of WT against the *otsA* and *glgA* mutants throughout the 10-day competition (Fig. 6A and B). Two-way ANOVA was performed on log_10_-transformed fold-change values to assess the interaction between time (Day) and carnosine treatment. Highly significant Day × Carnosine interactions were observed for all three mutant strains (*otsA*, *P* = 1.10 × 10^−13^; *glgA*, *P* = 1.22 × 10^−9^; *glgA otsA*, *P* = 4.44 × 10^−13^), demonstrating carnosine’s strong protective effect on competitive fitness.

Interestingly, carnosine supplementation did not significantly alter the competitive fitness of the *glgA otsA* double mutant against the WT strain up to day 5 (Fig. 6C). However, from day 5 to day 10, carnosine supplementation markedly reduced the competitive advantage of the WT strain, completely preventing the characteristic late-stage competitive collapse observed in the *glgA otsA* strain without carnosine supplementation and maintaining near-equal fitness with the WT.

### Disrupting glycogen or trehalose synthesis decreases ATP levels and redox activity

**Fig 7:**
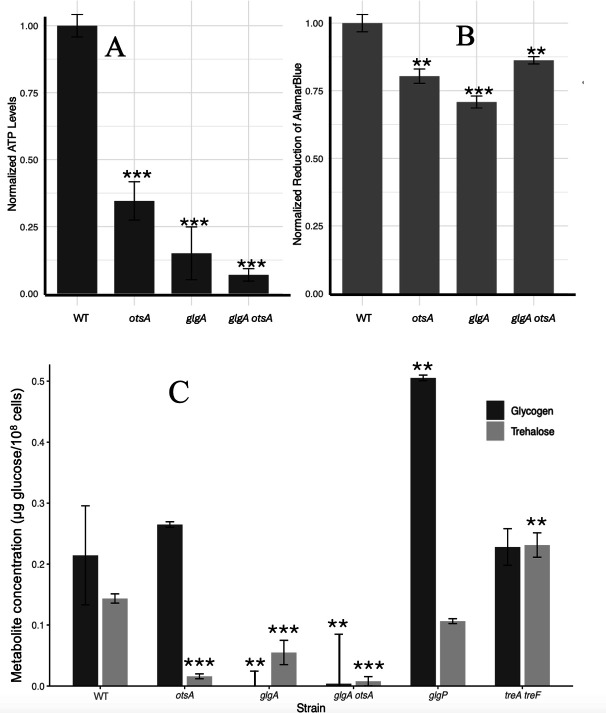
Energy metabolism and metabolite quantification. (**A**) ATP Assay of stationary phase cultures. Luminescence measurements were normalized to 1.0 for WT; *n* = 5 cultures per strain. (**B**) Redox activity of stationary phase cultures. AlamarBlue reduction measured by absorbance with measurements normalized to 1.0 for WT; *n* = 5 cultures per strain. (**C**) Glycogen and trehalose quantification of stationary phase cultures (16 h post-inoculation) in WT and mutant strains impaired in glycogen and/or trehalose synthesis or degradation; *n* = 3 cultures per strain. For all panels, statistical significance of mutant strain measurements compared to WT: **, *P* < 0.01; ***, *P* < 0.001; unpaired t-test; two-sided. Data are mean ± s.d.

ATP levels and metabolic activity were quantified in stationary phase cultures to study changes in energy metabolism. ATP levels were decreased for all mutant strains relative to WT, with the *otsA* strain showing higher concentration of ATP than the *glgA* strain; the *glgA otsA* double mutant strain exhibits the lowest ATP levels of any strain (Fig. 7A). Redox activity, as measured by the redox indicator AlamarBlue, was reduced in all three mutant strains relative to WT, with the *glgA* mutant strain more impaired than any strain (Fig. 7B). Interestingly, the *glgA otsA* double mutant strain showed increased redox activity relative to either the *otsA* or *glgA* mutant strains.

### Disrupting glycogen synthesis decreases trehalose abundance

To determine whether disrupting glycogen metabolism alters trehalose accumulation or vice versa, glycogen and trehalose levels were quantified during the stationary phase in all mutant strains (Fig. 7C). Glycogen was not detected in either the *glgA* or *glgA otsA* double mutant strains, which cannot synthesize glycogen, at 16 h post-inoculation, while glycogen over-accumulated relative to WT in the *glgP* mutant strain, which is unable to degrade glycogen. In the *otsA* and *treAF* mutant strains, which are unable to synthesize or degrade trehalose, respectively, glycogen levels were not perturbed. In the *otsA* and *glgA otsA* mutant strains, trace amounts of trehalose were detected, as previously reported (54), since trehalose is a component of LB medium (55). Over time, trehalose levels increased in the *treAF* mutant strain, unable to degrade trehalose, relative to WT. While trehalose levels were not significantly altered in the *glgP* mutant strain, the *glgA* mutant showed ~3-fold reduced trehalose accumulation relative to WT.

### The *glgA otsA* double mutant strain shows upregulation of the Crp regulon and downregulation of the Cra regulon

**Fig 8:**
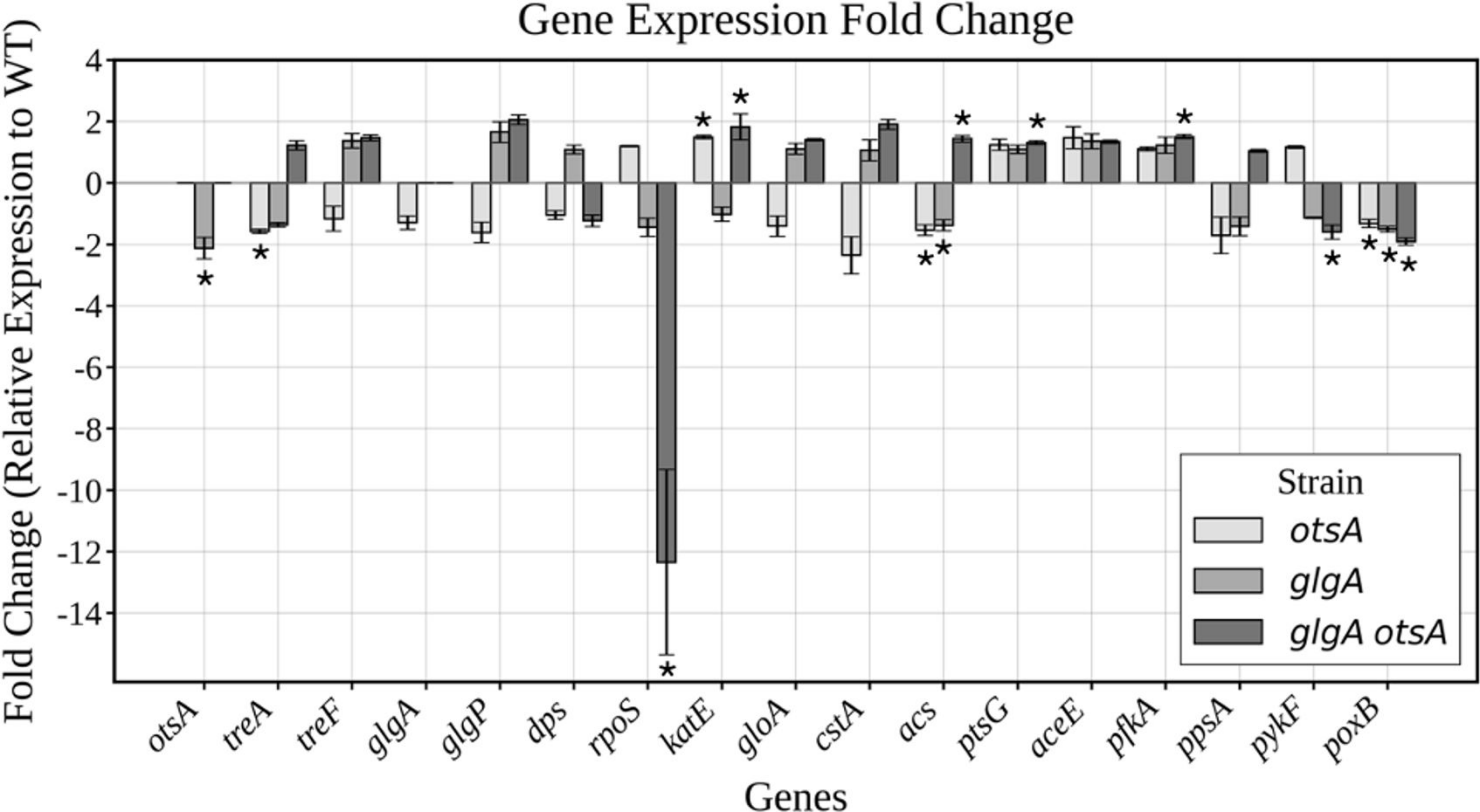
Gene expression changes in the *glgA otsA* strain relative to WT show CRP regulon upregulation and Cra regulon downregulation. qRT-PCR fold change analysis of mutant strains compared to the WT strain at 16 h. The *glgA otsA* double mutant strain displays increased CRP-regulated gene expression and decreased Cra-regulated gene expression. RT-PCR of mRNA from 16-h cultures, with fold change in gene expression relative to the WT strain; *n* = 3 cultures per strain (data are mean ± s.d.; *, *P* < 0.05; unpaired *t*-test; two-sided).

A *glgA otsA* double mutant strain is predicted to exhibit decreased glucose flux through the PTS due to the loss of trehalose recycling and experience increased glycolytic flux due to the inability to divert glucose into either glycogen or trehalose. Therefore, it was hypothesized that the *glgA otsA* double mutant strain would exhibit upregulation in cAMP-CRP and downregulation in Cra activity, corresponding with decreased glucose PTS flux and increased glycolytic flux, respectively. qRT-PCR was used to assess transcriptional changes during the stationary phase. Expression of genes encoding enzymes in glycogen or trehalose metabolism was quantified (Fig. 8). Of the glycogen and trehalose pathway genes tested in the mutant strains, only the gene *otsA* was differentially expressed, being downregulated in the *glgA* mutant strain. Differential expression of stress response genes was also assessed. *katE*, encoding the catalase HPII, was upregulated in the *otsA* and *glgA otsA* mutant strains, relative to the WT strain.

The *glgA otsA* double mutant strain displayed regulatory changes corresponding to upregulation of the cAMP-CRP regulon and reduction in Cra activity. Specifically, *rpoS*, the general stress response sigma factor that is negatively regulated by cAMP-CRP (56), was downregulated in the *glgA otsA* strain, relative to the WT strain. *ptsG* and *acs*, which are positively regulated by cAMP-CRP, were both upregulated in the *glgA otsA* strain. *pfkA*, encoding the glycolytic gene phosphofructokinase-1, which is negatively controlled by Cra, was upregulated, and the Cra-positively controlled genes *pykF* and *poxB*, encoding pyruvate kinase and pyruvate oxidase, respectively, were downregulated in the *glgA otsA* strain.

## DISCUSSION

Data from monoculture and coculture experiments demonstrate that loss of glycogen or trehalose synthesis compromises stationary-phase survival and reduces competitive fitness during both the stationary phase and LTSP. In addition, stress sensitivity and metabolic profiling indicate that trehalose primarily safeguards biomolecules, whereas glycogen maintains energy balance. Specifically, our experiments demonstrate that the *otsA* mutant exhibited increased sensitivity to heat and oxidative stress, while the *glgA* mutant displayed significantly reduced ATP levels and redox activity. Under high-glucose conditions, glycogen acted as a critical glucose buffer: WT cells sharply increased glycogen accumulation at 0.4% glucose, and glycogen-deficient strains showed reduced viability. This underscores the importance of glycogen as a glucose buffer under carbon excess.

Although glycogen and trehalose perform functionally distinct roles—glycogen is primarily associated with energy maintenance, and trehalose is primarily associated with stress protection—they partially compensate for one another. This functional specialization is evident in the mutant phenotypes: the *glgA* mutant shows severe ATP depletion and reduced redox activity, whereas the *otsA* mutant exhibits heightened sensitivity to heat and oxidative stress. However, the *otsA* mutant also exhibited energy deficits, and the *glgA* mutant showed heightened stress sensitivity, suggesting partial functional redundancy. Critically, the reduced trehalose accumulation observed in the *glgA* mutant (~3-fold decrease, Fig. 7C) suggests metabolic coupling between these pathways that has important implications for interpreting glycogen mutant phenotypes. Stress sensitivities attributed to glycogen deficiency in this and previous studies may partially reflect secondary effects on trehalose or other interconnected metabolic pathways. By measuring both glycogen and trehalose simultaneously in single- and double-mutants, our work reveals this coupling and provides a framework for disentangling the relative contributions of each pathway to bacterial stress tolerance and fitness. The asymmetric relationship between the glycogen and trehalose—where trehalose levels decline in the *glgA* mutant, but glycogen levels remain stable in the *otsA mutant*—implies that glycogen synthesis helps balance carbon allocation, including trehalose production. This finding is consistent with evidence from other organisms (such as cyanobacteria) showing that glycogen acts as a carbon sink that prevents metabolic imbalances and stabilizes central carbon metabolism (6, 51, 57). Together, these results reinforce the idea that carbon storage, coupled with appropriate flux through energy-spilling pathways, is critical for *E. coli* resilience and reveal the necessity of examining interconnected pathways to accurately attribute phenotypic effects.

How cells partition glucose-6-phosphate between glycogen and trehalose synthesis remains unclear, though the consistently greater flux toward glycogen may reflect enzyme kinetics, coordinated regulation by RpoS, or feedback from pathway products. The asymmetric relationship—where trehalose levels drop in the *glgA* mutant strain, but glycogen remains stable in the *otsA* mutant strain—suggests that glycogen synthesis exerts dominant control over carbon allocation between these pathways. However, trehalose synthesis appears less dependent on glycogen degradation. The *glgP* mutant displayed unchanged trehalose abundance. Overall, these results caution that phenotypes observed in glycogen-deficient mutants may partly reflect secondary effects on trehalose metabolism.

The phenotypes associated with the *glgA otsA double mutant* strain highlight the synergy between these pathways. This mutant exhibited marked sensitivity to heat, oxidative, and glycation stress, with elevated DCFH-DA fluorescence and accumulation of advanced glycation endproducts. The increased sensitivity to H₂O₂ and upregulated *katE* expression in the *otsA* and *glgA otsA* mutant strains provide evidence that oxidative stress contributes to the fitness defects in these strains. The higher growth yield at specific time points of the *glgA otsA* strain in 0.4% glucose, compared to WT and single mutant strains, suggests increased allocation of excess carbon to biomass in the absence of both glycogen and trehalose synthesis. This diversion of excess carbon to biomass likely represents dysregulated vegetative rather than adaptive reallocation, as wild-type cells use glycogen and trehalose as carbon sinks to prevent inappropriate biosynthesis when growth is metabolically unfavorable. Without these storage pathways, the *glgA otsA* double mutant channels excess carbon into biosynthetic processes that generate toxic byproducts (elevated ROS and AGEs) and deplete ATP reserves, ultimately compromising long-term survival despite short-term competitive gains. Together, these results reveal that glycogen and trehalose cooperatively buffer high-carbon stress and external challenges. The double knockout also showed negative epistasis—stress sensitivity greater than either single mutant—indicating functional interdependence between the two pathways.

Interestingly, despite its stress vulnerability, the *glgA otsA* strain displayed a transient competitive advantage against WT. This pattern suggests a tradeoff between stress resistance and nutrient scavenging. Although the double mutant exhibited the lowest ATP pool levels, it showed elevated redox activity, as measured by the AlamarBlue assay. Combined with transcriptional data, these findings indicate modulation of the global regulators cAMP-CRP and Cra, driving enhanced carbon scavenging and glycolytic flux.

The competitive benefit of the *glgA otsA* mutant strain against WT early during LTSP, coupled with the fitness deficits displayed by the single knockout strains, suggests a compensatory mechanism induced in the double knockout strain. Enhanced fitness in LTSP often reflects tradeoffs in survival strategy, as exemplified by the Growth Advantage during Stationary Phase (GASP) phenotype, in which mutations in the master stress response regulator *rpoS* reduce stress tolerance, but enhance nutrient utilization (38, 58). Similarly, the double knockout’s stress sensitivity and short-term competitive advantage reflect a metabolic strategy that favors elevated resource acquisition at the expense of stress resilience (59, 60). *This* trade-off likely arises from global regulator imbalances, in which loss of carbon storage elevates glycolytic flux and cAMP–CRP signaling while reducing Cra activity. Interestingly, the *glgA otsA* mutant showed reduced *rpoS* expression, possibly mediated by increased activation of Crp, which can downregulate *rpoS* (56).

Glycogen and trehalose have long been recognized as key contributors to bacterial adaptability and resilience (61–63). The data reported here expand this understanding and highlight the versatile physiological roles of both carbohydrates. Trehalose—generally considered solely as an osmoprotectant and stress metabolite in *E. coli*—also supports energy maintenance and metabolic regulation (8). Likewise, glycogen, once regarded as a passive carbon reserve, functions as a dynamic carbon store that modulates carbon flux, drives metabolic transitions, and facilitates growth resumption (64, 65). Our results expand this functional repertoire and show that glycogen and trehalose act independently and synergistically. This relationship optimizes carbon homeostasis, with the integration of both pathways and global regulators to balance the cellular energy budget and reprogram metabolism under changing conditions.

**Fig 9:**
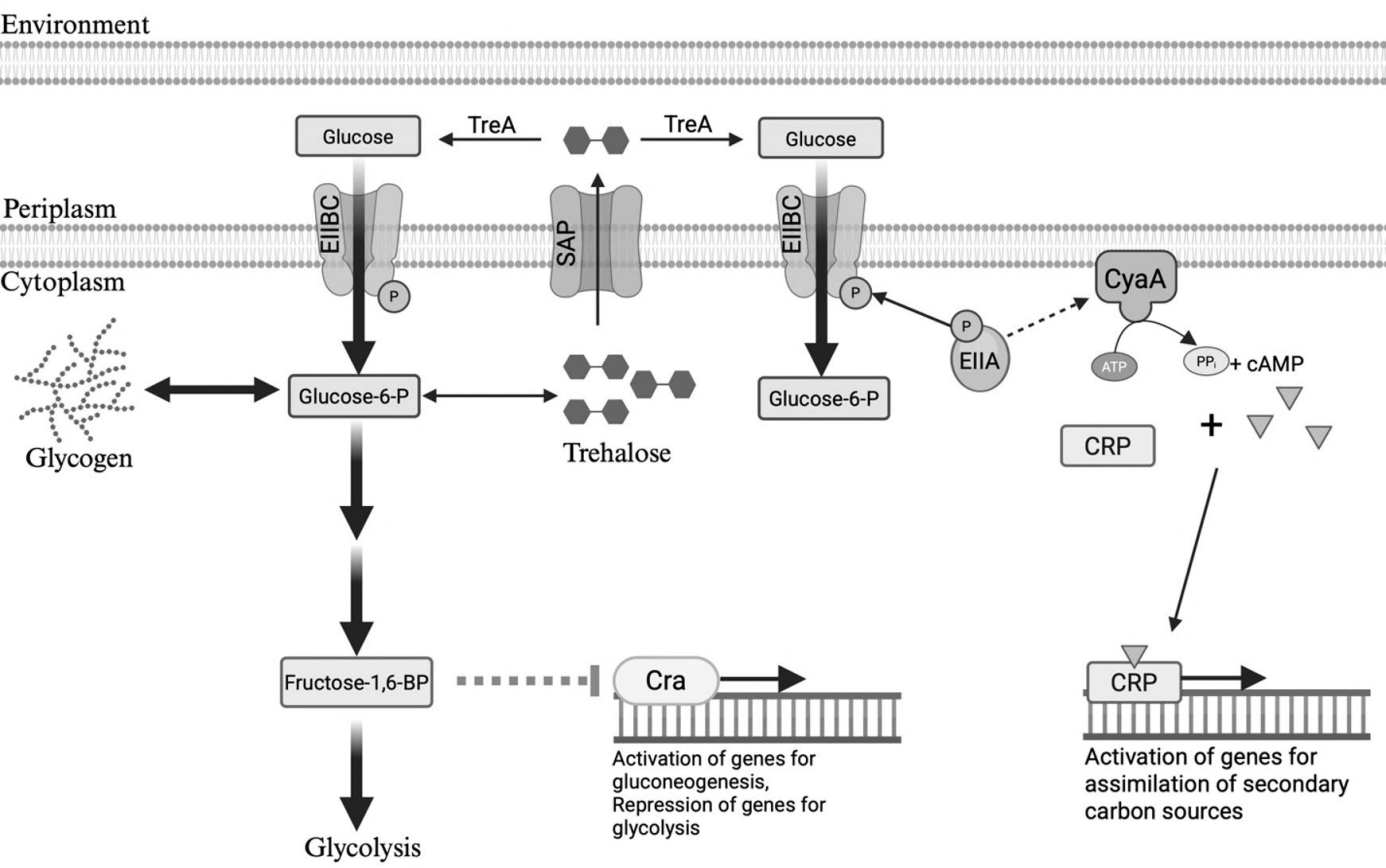
Model linking trehalose recycling, glycogen metabolism, and global regulation during the stationary phase. During nutrient depletion, *E. coli* coordinates trehalose and glycogen metabolism with the global regulators CRP–cAMP and Cra to balance energy flux, carbon storage, and stress resilience. Periplasmic trehalose recycling sustains moderate glucose import through the PTS, moderating CRP activity. Glycogen serves as the predominant intracellular carbon reserve, storing a substantially greater fraction of glucose equivalents than trehalose and buffering excess carbon during nutrient-rich periods. In contrast, trehalose acts as a smaller but rapidly mobilizable pool that also protects biomolecules and modulates signaling. Loss of trehalose synthesis or recycling (*otsA*) decreases PTS flux, elevating CRP activity and reducing RpoS-dependent stress tolerance. Loss of glycogen synthesis (*glgA*) prevents glucose sequestration, increases glycolytic flux, and lowers Cra activity. In the *glgA otsA* double mutant, simultaneous disruption of both pathways exaggerates these effects, producing elevated CRP activation, diminished Cra control, and hyperglycolytic metabolism that leads to ROS accumulation, ATP depletion, and protein glycation. Together, glycogen and trehalose form interconnected carbon buffers linking glucose flux and storage to global regulatory networks, coordinating the metabolic transitions that define stationary-phase adaptation in *E. coli*.

Trehalose and glycogen metabolism both draw from and feed into central carbon metabolism through their shared precursor, glucose-6-phosphate, allowing them to act as carbon sinks that regulate flux through glycolysis. Previous work showed that disruption of periplasmic trehalose recycling perturbs cAMP–CRP signaling, which governs uptake and catabolism of alternative carbon sources (8). Trehalose periplasmic recycling may function in what appears to be a futile cycle. The simultaneous export of trehalose and energy-dependent import of the liberated glucose expends energy seemingly wastefully while also influencing metabolic programming via moderating CRP activity (10). Accordingly, depletion of both trehalose and glycogen dysregulates cAMP–CRP and Cra activity, triggering compensatory upregulation of transporters (PtsG) and catabolic enzymes (Acs) to maximize short-term nutrient acquisition (Fig. 9). This strategy is advantageous in the short term to enhance immediate responses to changing substrate availability. However, this tradeoff ultimately compromises stress defense and may be detrimental to long-term survival.

Collectively, these findings illuminate a previously underexplored connection between nutrient storage and global regulatory networks in *E. coli*. Glycogen and trehalose potentially function as integrated modulators of cAMP-CRP and Cra, aligning metabolic activity with carbon availability. Glycogen is increasingly recognized as a dynamic carbon reserve that supports stress resistance and metabolic flexibility, while trehalose emerges as a key regulator of energy balance and metabolic signaling. The close temporal coordination of glycogen and trehalose synthesis and degradation during the stationary phase indicates a high degree of regulatory integration. Furthermore, both the glycogen and trehalose pathways may continuously fine-tune transcriptional programming in response to shifting carbon availability. Future studies should explore how these temporal changes correlate with intracellular metabolic and transcriptional rewiring as cells adapt to energy limitation. Finally, these insights advance a growing effort to untangle the complex crosstalk between transcriptional and metabolic networks that optimize bacterial fitness under different environmental and stress conditions. Altogether, the redundancy between glycogen and trehalose may confer a selective advantage under ever-changing environments, improving overall survivability.

## SUPPLEMENTAL MATERIAL

Supplemental figures jb.00544-25-s0001.pdf Figures S1 and S2.

ASM does not own the copyrights to Supplemental Material that may be linked to, or accessed through, an article. The authors have granted ASM a non-exclusive, world-wide license to publish the Supplemental Material files. Please contact the corresponding author directly for reuse.
