# Supplementary material for: Synergistic interactions between glycogen and trehalose mediate adaptation to the stationary phase in E. coli
Source: J Bacteriol. 2026 Apr 22;208(5):e00544-25. doi: 10.1128/jb.00544-25 (PMC13192265; doi:10.1128/jb.00544-25)
Supplement: Supplemental figures — Figures S1 and S2. [file jb.00544-25-s0001.pdf]

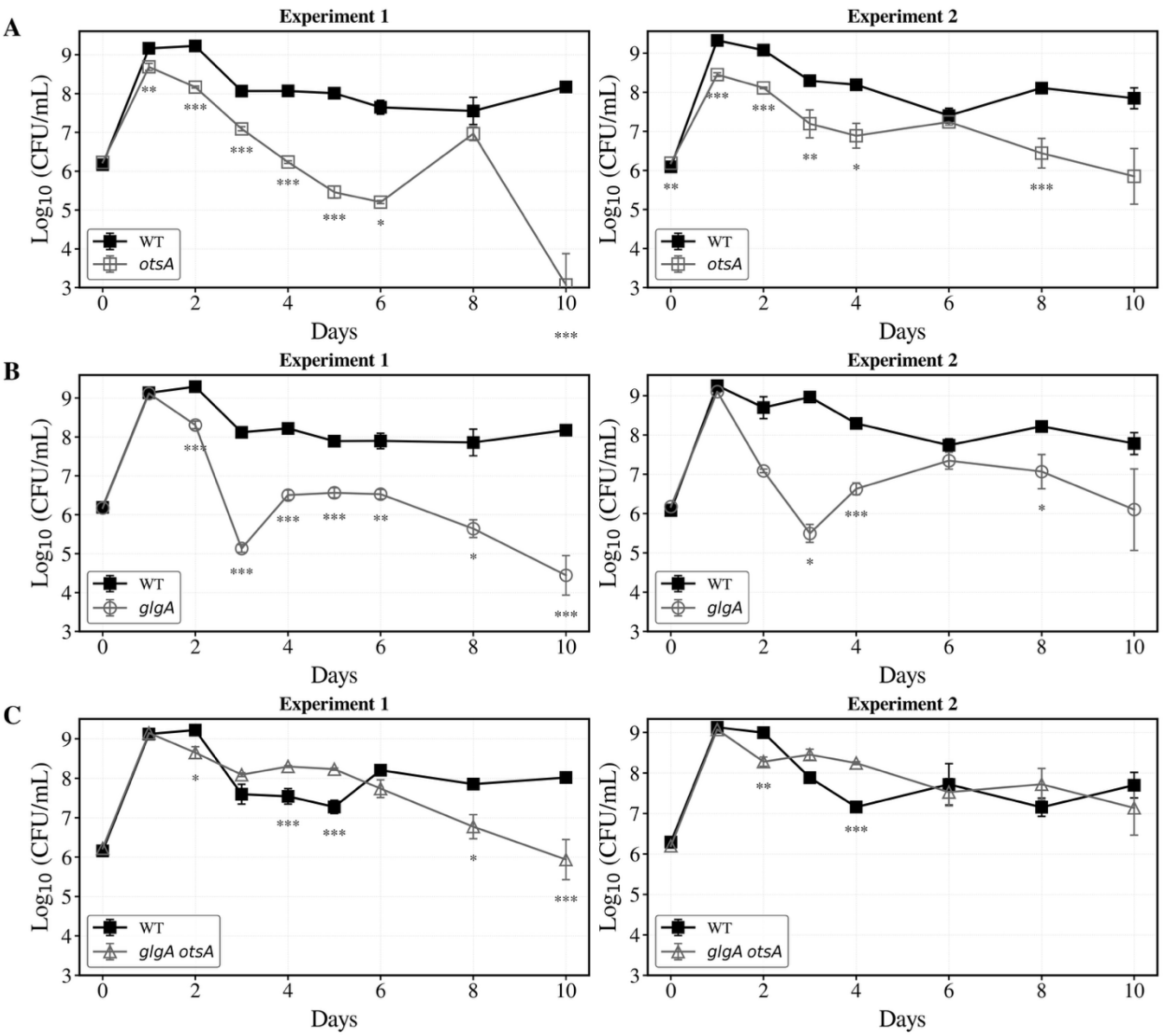

Supplementary Figure S1. Competitive fitness phenotypes in independent experiments. Competition between WT and glycogen/trehalose synthesis mutants in LB medium over 10 days. Two additional independent experiments (see representative data in Figure 3) shown side by side for each mutant: Experiment 1 (left, n=5 biological replicates) and Experiment 2 (right, n=3 biological replicates). Black filled squares, WT; dark gray hollow markers, mutants: (A) *otsA* (squares), (B) *glgA* (circles), (C) *glgA otsA* (triangles). Data are mean ± s.d. Statistical significance of mutant strain viability is compared to WT at each timepoint: *, $P < 0.05$; **, $P < 0.01$; ***, $P < 0.001$; unpaired t-test; two-sided.

Reproducible findings: (1) *otsA* shows consistent Day 1 fitness deficit and sustained competitive disadvantage. (2) *glgA* exhibits reproducible Day 2-3 competitive impairment. (3) *glgA otsA* displays consistent biphasic dynamics with early competitive advantage (Days 3-5) followed by late decline (Days 6-10). Results confirm reproducibility of competitive phenotypes across independent biological experiments.

A

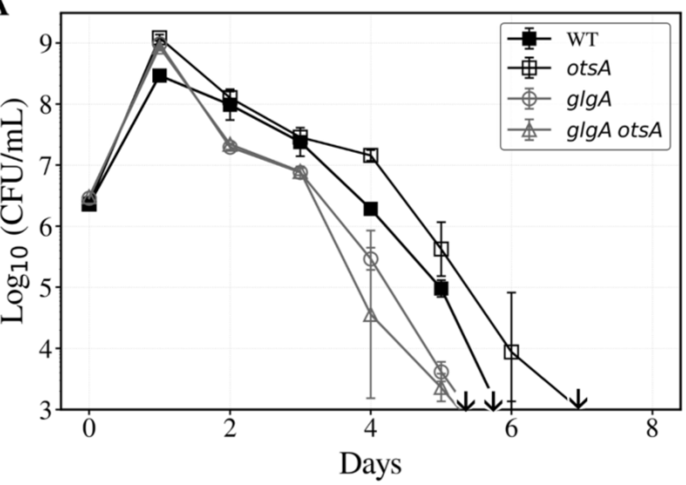

Day	*otsA*	*glgA*	*glgA otsA*
0	ns	ns	*
1	**	*	**
2	ns	ns	ns
3	ns	ns	ns
4	*	*	*
5	ns	*	*
6	ns	ns	ns

B

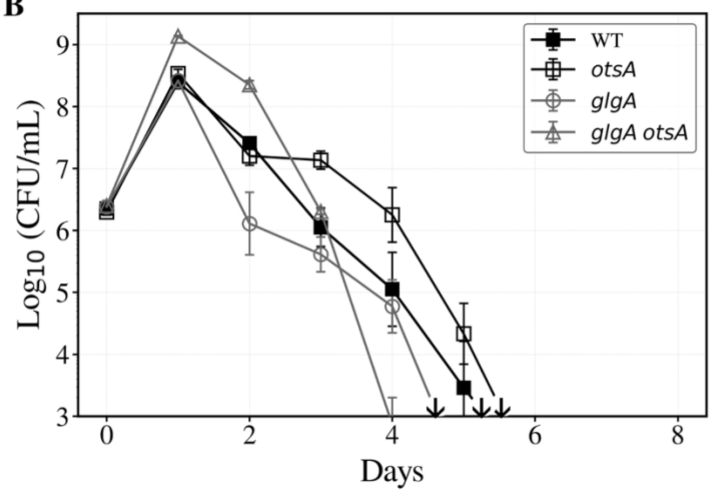

Day	*otsA*	*glgA*	*glgA otsA*
0	ns	ns	ns
1	ns	ns	***
2	ns	**	**
3	*	ns	ns
4	ns	ns	ns
5	ns	ns	ns
6	ns	ns	ns

Supplementary Figure S2. Glucose stress phenotype in independent experiments. Survival of WT and glycogen/trehalose synthesis mutant strains in 0.4% glucose-supplemented LB medium across two independent experiments (A, B; n=3 per experiment). Two additional independent experiments (see representative data in Figure 5B) shown. Left: Log_{10} CFU/mL over 8 days (mean ± s.d.) (↓= below limit of detection, 10^3 CFU/mL). Right: Statistical significance (unpaired t-test, Days 0-6; *, $P < 0.05$; **, $P < 0.01$; ***, $P < 0.001$; ns, not significant). Black filled squares, WT; black hollow squares, *otsA*; dark grey hollow circles, *glgA*; dark grey hollow triangles, *glgA otsA*. Both experiments show: (1) *glgA otsA* increased cell density yields at Day 1; (2) *otsA* enhanced survival Days 1-4; (3) *glgA* and *glgA otsA* increased sensitivity Days 4-6.
